# Pregnancy Outcomes After Renal Transplantation: A Retrospective Case Series

**DOI:** 10.1089/whr.2019.0002

**Published:** 2020-02-10

**Authors:** Jiang Ying, Lin Li, Yan Zhai, Shuzhen Wang, Xiaobei Li

**Affiliations:** ^1^ Department of Obstetrics and Gynecology, Chaoyang Hospital Affiliated to Capital Medical University, Beijing, China.; ^2^ Department of Urinary Surgery, Chaoyang Hospital Affiliated to Capital Medical University, Beijing, China.

**Keywords:** renal transplantation, pregnancy, pregnancy outcome, pre-eclampsia, renal function

## Abstract

***Objective:*** To investigate the clinical characteristics and outcomes of pregnancies after renal transplantation (RT).

***Materials and Methods:*** A retrospective study was employed. Data of obstetric, transplantation, pregnancy outcomes, and maternal and neonatal complications from 2000 to 2017 were obtained from a single obstetrics center.

***Results:*** Eleven cases of pregnancies were included: the mean age at conception was 31.27 ± 3.44 (26–36, median 32) years and interval from RT to pregnancy was 51.18 ± 30.65 (25–132, median 38) months. The nine successful pregnancies (9/11) were all in women who maintained their immunosuppressive regimens. All infants were delivered by cesarean section for severe pre-eclampsia in eight cases and placental abruption in one case at a mean gestational age of 34.67 ± 1.41 (30–38) weeks and a median birth weight of 2374.56 ± 569.00 (1,325–3,070) g. Four of nine infants had low birth weight, and six of nine infants underwent premature delivery. The babies had no postpartum complications or congenital anomalies at follow-up period (12–180 months, mean 98.18 ± 54.73 months). One infant was transferred to the neonatal intensive care unit for neonatal asphyxia. Nine cases were uncomplicated at the first trimester, but developed severe pre-eclampsia after the second trimester. Two patients had allograft dysfunction from the second trimester and delivered at the third trimester, and then lost the allografts at 2 and 6 years postdelivery, respectively, whereas the other seven patients had well-functioning allografts at an average follow-up of 108 months. Two patients who decreased their immunosuppressive regimens and developed severe complications had second trimester therapeutic abortion and lost their allografts

***Conclusions:*** During pregnancy, RT patients exhibit risks for the mother, fetus, newborn, and allograft. Decreasing immunosuppressors leads to poor outcomes. With proper peritransplant and periconceptional counseling, these patients can have acceptable pregnancy outcomes.

## Introduction

Reproductive function is impaired in patients with severe renal insufficiency. Most of these patients suffer from chronic anovulation and subsequently delayed conception. After transplantation, renal and endocrine functions rapidly recover, and pregnancy becomes possible. Pregnancy after renal transplantation (RT) is considered a high-risk pregnancy, associated with increased risks of pre-eclampsia, preterm delivery, fetal growth restriction (FGR), and graft rejection, among other issues. The influence of immunosuppressants on the mother and fetus is also an important consideration. For these pregnancies, collaborations among obstetricians, nephrologists, urologists, and pediatricians are required throughout the journey of pregnancy and labor.

Many pregnancies after RT have been reported; however, there are limited data on the optimal counseling and clinical management of these patients. The pregnancy does not typically show a severe impact on the renal graft function in the short term; however, the long-term impact is less clear. We present our experience with 11 renal allograft recipients over the past 18 years. The prepregnancy and postdelivery renal function, outcome of gestation, and maternal and fetal complications were retrospectively assessed. In this study, we evaluated the effect of pregnancy on the kidney-recipient mother, the newborn, and the graft.

## Materials and Methods

This study is a retrospective observational study conducted in the Department of Obstetrics of Chaoyang, from January 2000 to December 2017; there were 13 pregnant patients who had undergone RT.

Two of the 13 pregnancies were unplanned and were electively terminated and excluded from the analysis.

In this series, RTs had been performed at different hospitals in Beijing. The following data were obtained: maternal age at pregnancy, interval between transplantation and conception, parity, allograft type, immunosuppressive therapy, associated hypertension, pregnancy duration at the first prenatal visit, pregnancy outcomes, and postdelivery renal function.

### Methods

Maternal and fetal outcomes were assessed, and the mother and baby were followed until December 31, 2017

#### Inclusion criteria

For inclusion, the patients should have optimal kidney function, no proteinuria or hypertension, and no evidence of the occurrence of graft rejection. In patients who meet these criteria, a decision can be made to become pregnant after 2 years post-transplantation.

#### Exclusion criteria

Patients who were unmarried, divorcee, and who had unplanned pregnancies were excluded.

### Definitions

Arterial hypertension refers to the presence of a systolic arterial pressure of 140 mm Hg, a diastolic pressure of 90 mm Hg, or the need for antihypertensive drugs. Pre-eclampsia refers to a new onset of hypertension and proteinuria, end-organ dysfunction or both after 20 weeks of gestation in a previously normotensive woman; severe hypertension and signs/symptoms of end-organ injury are considered the severe spectrum of the disease.

Abortion refers to the termination of gestation before week 28, demise refers to the termination of gestation after 28 weeks of pregnancy with a dead newborn, neonatal death refers to a newborn who dies before reaching 28 days of age, and maternal death refers to a mother who dies during gestation or the postpartum period.

Preterm delivery refers to the end of the pregnancy before reaching 37 weeks of gestational age. Low birth weight (LBW) refers to a newborn who weighs 2,500 g, regardless of the gestational age.

RT patients were evaluated by obstetricians and nephrologists and met the previously described conditions. During the first prenatal visit, blood pressure was measured and general laboratory tests, including the complete blood cell count, basic metabolic profiles, urine assay, and blood group verification, were performed. An ultrasonographic examination was also performed. Results from the prepregnancy renal function tests were obtained from the referring institution, and results from the predelivery renal function tests were obtained from case notes at the time of delivery admission. Complications during pregnancy were reviewed, as well as pregnancy duration at delivery, mode of delivery, indication for operative delivery (forceps or cesarean delivery), and maternal and baby need for hospitalization in the intensive care unit.

Routine antenatal visits to high-risk pregnancy clinics were arranged monthly during the first half of pregnancy, fortnightly until the 32nd week, and then weekly unless certain indications indicated more frequent visits. At each visit, all women were assessed for a full blood count, blood urea, serum creatinine and creatinine clearance, serum electrolytes, urine analysis, and maternal renal ultrasound scanning. Routine ultrasound scans were performed at early pregnancy and at the 13th, 18th, 24th, and 37th weeks of gestation, respectively.

Neonatal outcome was determined from birth weight and length, Apgar scores at 1 and 5 minutes, gestational age, and intrauterine growth restriction as determined by pediatricians after delivery based on neonatal and maternal characteristics. Whether the newborn required treatment in the neonatal intensive care unit (NICU) or was discharged with the mother was also determined, as well as the presence of congenital anomalies.

Follow-up was conducted at regular intervals and included maternal renal ultrasound scanning. Routine ultrasound scans and the baby's physical and intellectual development were examined.

### Statistical analysis

The values are expressed as the absolute frequency and the mean and median with the interquartile range. The count data are expressed as a percentage. The significance value was *p* < 0.05. Data analysis was performed using the SPSS statistical package for Windows.

## Results

### Patient characteristics

All pregnancies after RT were registered during the analyzed period and included 13 recipients of RT. The age at the time of conception was 31.27 ± 3.44 (range 26–36, median 32) years. The time interval from transplantation to conception in the planned pregnancies was 51.18 ± 30.65 (range 25–132, median 38) months. Table 1 summarizes the clinical and analytic results at the time of the first consultation after pregnancy was diagnosed. The patients had no prior pregnancy or delivery post-RT.

**Table 1.** Clinical and Analytical Data at Different Time Points of Gestation and Postpartum

	Prepregnancy *n/N*	First trimester	Second trimester^a^	Third trimester	Postpartum
Immunosuppressive regimens unchanged	11/11	9/11	9/11	9/9	9/9
Creatinine (mg/dL) (*n*/*N*)	1/11^b^	2/11	3/11	3/9	2/9
Proteinuria (mg/L) (*n*/*N*)	0/11	2/11	4/11	9/9	2/9
Arterial pressure (mm Hg) (*n*/*N*)	0/11	2/11	4/11	9/9	3/9

*N* indicates the total cases and *n* indicates abnormal cases.

^a^ Two cases decreased the immunosuppressive regimens and required termination of the pregnancy during the second trimester; 9 cases were discussed from the second trimester.

^b^ The serum creatinine level was not recorded in one case.

### Graft function

The serum creatinine levels before pregnancy were less than 1.0 mg/dL in all cases with the exception of one case whose data were not available. The mean serum creatinine level at the time of the first prenatal visit was 0.9 ± 0.1 (median 1.0 mg/dL; range: 95% CI 0.6–1.1). In nine cases in whom the immunosuppressive regimens were not changed during pregnancy, the creatinine levels were normal during the first trimester. Two patients had an impaired allograft function at 24 and 34 weeks and cesarean section (CS) delivery was accomplished at 30 and 36 weeks, respectively. Six patients had well-functioning allografts during pregnancy, another patient had impaired allograft function with return to normal postpartum. All of nine patients were followed up for 12–180 months.

The nine cases who continued the same immunosuppressive drugs during prepregnancy and pregnancy had negative or minimal proteinuria during the first and second trimesters and gradually accumulated large amounts of proteinuria at 24–36 weeks of gestation, with a quantitative amount of 625–12,555 mg per 24 hours; six cases returned to negative or minimal proteinuria after delivery, and three cases were consistently positive for urinary protein 1 year after delivery. Two patients who had allograft dysfunction at 24 and 34 weeks ultimately lost the allografts after 2 and 6 years, respectively.

### Pregnancy outcomes

None of the 11 planned pregnancies was ectopic. Nine of 11 (81.81%) pregnancies were successful and were all delivered by CS. The mean gestational age in the nine cases who maintained the immunosuppressive regimens was 34.67 ± 1.41 (range 30–38) weeks. Although four of nine infants exhibited LBW, their median birth weight was 2374.56 ± 569.00 (range 1,325–3,070) g. Premature delivery occurred in six live births. There were no postpartum complications with the newborns with the exception of one baby. The baby was transferred to the NICU due to neonatal asphyxia, preterm delivery, and early gestational age, was hospitalized in the NICU for 16 days, and was discharged home with a stable status (Table 2).

**Table 2.** Evolution of Pregnancies: Maternal and Fetal Complications

Number of patients (*n*)	11
Number of pregnancies (*n*)	11
Duration to pregnancy (month, mean ± SD)	51.18 ± 30.65 (range 25–132)
Gestational age when born (weeks, mean ± SD)	34.67 ± 1.41 (range 30–38)
Live births (*n*)	9
Abortions (*n*)	2
Ended in CS (*n*)	9
Birth weight (g, mean ± SD)^a^	2374.56 ± 569.00 (range 1,325–3,070)
LBW (*n*)	4/9
Premature delivery (*n*)	6/9
Hypertension pre-eclampsia/eclampsia (*n*)	11
Living/deceased donor (*n*)	7/9

CS, cesarean section; LBW, low birth weight; SD, standard deviation.

^a^ including the 9 cases of successful CS.

At an average follow-up of 108 (range 12–180, mean 98.18 ± 54.73) months postdelivery, all nine babies were normal, and no congenital anomalies were documented.

### Maternal complications

Of the 11 planned pregnancies, 9 cases who maintained immunosuppressive therapy had no associated complications in the first trimester; however, all cases suffered from severe pre-eclampsia in the second and third trimesters. They were treated following the standard protocol designed for hypertensive disease in pregnancy. The pregnancies ended with a successful CS at 30–38 weeks. The indication for operative delivery included severe pre-eclampsia, as well as one patient who suffered from the placental abruption and fetal anoxia and the pregnancy was terminated with an emergency cesarean section at 30 weeks. Urinary tract infections, prelabor rupture of membranes, and diabetes mellitus were not noted in these patients (Table 2).

The other two patients who decreased the immunosuppressive regimens suffered from severe complications, including severe pre-eclampsia, HELLP (hemolysis, elevated liver enzymes, and low platelets) syndrome, heart failure, severe anemia, and renal failure at 17 and 19 weeks, respectively. The pregnancies were terminated with induced abortion.

## Discussion

### Gestational complications in RT patients

There have been many reports of successful pregnancies in women with RT. With an increasing number of successful renal transplants being performed in women of childbearing age and a sizeable cohort of pediatric kidney transplant recipients approaching adulthood, significant data have been accumulated regarding pregnancy outcomes in renal transplant recipients. However, pregnancy in allograft recipients is associated with unique antepartum, intrapartum, and postpartum problems that require special management. Pregnancy in these women involves risks to the mother, graft, and fetus. The most common pregnancy complications in these patients include infection, pre-eclampsia, premature delivery, premature membrane rupture, intrauterine growth retardation, LBW, gestational diabetes, and graft rejection.^1–3^ In our study, all patients suffered from pre-eclampsia, which is related to premature delivery, intrauterine growth retardation, LBW, and neonatal complications. Severe pre-eclampsia affected postnatal renal function, and two patients lost their grafts at 2 and 6 years after delivery, respectively; moreover, the incidence of pre-eclampsia was higher than reported in previous studies.^1–6^ From the largest registers, the incidence of hypertension during pregnancy has varied between 58% and 72% in all kidney post-transplantation pregnancies.^4^ Ghafari and Sanadgol^5^ reported an incidence of 40%.^5^ Wielgos et al.^7^ reported 89.5% of pregnancy-induced hypertension after RT.^7^ The relationship between RT and pre-eclampsia is unclear; however, it should not be related to the transplantation, as the incidence of gestational hypertension after liver transplantation is significantly lower than RT.^8^ It may also be that some characteristics of the transplanted kidney are associated with the risk of pre-eclampsia.

In addition to certain complications, kidney transplant recipients are also at a higher risk for ectopic pregnancy because of previous surgical procedures and continuous ambulatory peritoneal dialysis. However, none of the nine pregnancies in our series were ectopic. Although pregnancy in graft recipients is not regarded as an indication for cesarean delivery, all late pregnancies after RT were delivered *via* cesarean section in our study. The most common indication for a cesarean section in our analyzed data included pre-eclampsia; it occurred more often than in the data reported by the NTPR (National Transplantation Pregnancy Registry)^4^ and was related to the higher incidence of pre-eclampsia. All premature deliveries were iatrogenic rather than spontaneous preterm labor, and iatrogenic premature deliveries were also related to the higher incidence of pre-eclampsia.

### Graft function

Most studies suggest that pregnancy is not consistently associated with graft deterioration.

In our study, in 2 of 11 cases, the renal function worsened from the second trimester, and the grafts were lost at 2 and 6 years after delivery, respectively. In our study, all pregnancies after RT suffered from severe pre-eclampsia, which may lead to the decline in renal function. New-onset hypertension from pregnancy certainly could have contributed to the decline in renal function and has a greater impact on transplanted kidneys. Thus, it is essential to achieve normal function during the preconception period for RT patients. According to the European Best Practice Guidelines IV, the conditions that are considered to be satisfactory obstetric results in patients after kidney transplantation are as follows: optimal kidney function, no proteinuria or hypertension, and no evidence of the occurrence of graft rejection. In patients who meet these criteria, a decision can be made to become pregnant after 2 years post-transplantation. From our data, patients who maintained immunosuppressive therapy during pregnancy had good pregnancy outcomes; however, two patients lost their grafts after 2 and 6 years, respectively. As the two patients who decreased or discontinued the immunosuppressive regimen had poor outcomes with loss of allograft function and required termination of pregnancy, it is critical to provide prepregnancy counseling and health care for women of RT.

### Pregnant outcome

The impact of pregnancy on kidney graft function and the effects of immunosuppressive drugs on fetal development remain unclear. None of the nine babies in our series had congenital malformations or immunological disease. However, six of nine cases had premature delivery, and four of nine cases had a LBW because of severe pre-eclampsia. Renal transplant recipients should be comanaged by a multidisciplinary team involving maternal fetal medicine specialists and transplant nephrologists. Because pregnant renal transplant recipients are at significantly increased risk for maternal and fetal complications, prenatal visits should occur more frequently in this population. It is necessary to individually assess each pregnancy.^9^ If the function of the transplanted kidney is stable before pregnancy, the patients may have an acceptable outcome. In our series, the function of the transplanted kidney was stable when the immunosuppressive therapy was not changed during pregnancy.

We conclude that although pregnancy in renal transplant recipients has high risks, complications from pre-eclampsia occurred in all patients, and premature delivery by CS was common. Fortunately in this series, neonatal outcomes were acceptable. This outcome is particularly achieved when the graft function is stable and when the post-transplant interval is >2 years.

## Abbreviations Used

CS: cesarean section
LBW: low birth weight
NICU: neonatal intensive care unit
RT: renal transplantation
SD: standard deviation
